# Hyperleukocytosis in a Patient With Chronic Myeloid Leukemia and HIV: A Case Report

**DOI:** 10.7759/cureus.100700

**Published:** 2026-01-03

**Authors:** Javier B Chambi-Torres, Adriana Penate Armesto, Temilola O Majekodunmi, Sohair Angly, George Michel

**Affiliations:** 1 Internal Medicine, Larkin Community Hospital, South Miami, USA

**Keywords:** chronic myeloid leukemia, dasatinib, hiv, hydroxyurea, hyperleukocytosis

## Abstract

The coexistence of chronic myeloid leukemia (CML) and human immunodeficiency virus (HIV) presents a unique clinical challenge due to potential drug interactions and compounded side effects. This case report discusses a 55-year-old male with HIV and poorly managed CML, who presented with severe hyperleukocytosis. Potential leukostasis was managed using leukapheresis, hydroxyurea, and continued antiretroviral therapy before initiating dasatinib (a tyrosine kinase inhibitor), given its therapeutic effect on CML and potential properties to target HIV infection. This approach underscores the importance of rapid intervention, cytoreduction, and careful selection of therapy to minimize complications. Multidisciplinary coordination allowed for successful management, balancing leukemia and HIV treatment needs. This case emphasizes the complexity of CML-HIV co-treatment and the importance of tailored therapy, highlighting the need for further research on dual therapy impacts to improve outcomes and quality of life in co-infected patients.

## Introduction

The coexistence of chronic myeloid leukemia (CML) and human immunodeficiency virus (HIV) infection presents a unique and complex clinical challenge that has been rarely reported. Each condition requires targeted treatment, which needs careful coordination, including review for potential drug-drug interactions and overlapping toxicities. 

The coexistence of CML and HIV is most likely coincidental [1]; the adherence to antiretroviral therapy (ART) in people living with HIV (PLWH) has improved overall survival and reduced the incidence of AIDS-defining malignancies [2], like Kaposi sarcoma and human papillomavirus (HPV) cervical cancer. However, increased HIV survival means that PLWH may develop other types of cancer and other comorbid conditions, including an increased incidence of non-AIDS-defining cancers [3].

CML is a myeloproliferative neoplasm that is characterized by uncontrolled proliferation of myeloid cells with abnormal tyrosine kinase activity from the translocation between the breakpoint cluster region (BCR) gene on 22q11.2 and the Abelson murine leukemia (ABL1) gene on 9q34.1 [4] with tyrosine kinase inhibitors (TKIs) as the main treatment. Hyperleukocytosis, defined as white blood cell (WBC) count >100,000/µL [5], can be a sign of uncontrolled CML [6]. 

We present a case of a 55-year-old male PLWH and uncontrolled CML who presented with hyperleukocytosis and was successfully treated with a single session of leukapheresis, daily hydroxyurea, allopurinol, and multi-specialty management.

## Case presentation

A 55-year-old Hispanic male, diagnosed with HIV in 2006 and currently adherent to a regimen of bictegravir, emtricitabine, and tenofovir alafenamide (TAF), presented with severe leukocytosis (white cell count of 429 x 10^3^/uL as per outpatient documentation) found during a recent outpatient medical evaluation in 2024. His medical history includes CML, diagnosed four years prior, but left untreated for several years due to poor adherence, as well as two previous myocardial infarctions at ages 25 and 35 years old (extent of work-up unknown by the patient), ocular melanoma, and gout. On admission, he complained of dizziness, weakness, unilateral right-sided headache, blurry vision in his left eye, shortness of breath on ambulation, palpitations, and upper back pain. His initial laboratory results revealed leukocytosis, with a WBC of 317 x 10^3^/uL, normal electrolyte levels, and other laboratory findings (Table 1).

**Table 1:** Laboratory results

Hematology	Result	Reference range
White blood cells	317.07 x 10^3^/uL	5.87-11.5 10^3^/uL
Helper T-lymphocyte CD4	1600 cells/mm^3^	500-1500 cells/mm
Hemoglobin	9.6	12.1-16.1 d/dL
Hematocrit	27.0	36.0-47.7 %
Mean corpuscular volume	90.3	79.0-92.2 fL
Platelets	209.0	150-450 10^3/uL
Mean platelet volume	11.2%	8.8-12.4 fL
Neutrophils	50.4%	34.0-67.9 %
	159.73 x 10^3^/mcL	1.78-5.38 x 10^3^/mcL
Lymphocytes	2.8%	21.8-53.1 %
	8.93 x 10^3^/mcL	1.32-3.57 x 10^3^/mcL
Monocytes	3.4%	5.3-12.2 %
	10.89 x 10^3^/mcL	0.30-0.82 x 10^3^/mcL
Eosinophils	1.7%	0.8-7.0%
	5.26 x 10^3^/mcL	0.04-0.54 x 10^3^/mcL
Basophils	4.5%	0.2-1.2%
	14.38 x 10^3^/mcL	0.01-0.08 10/mcL
Chemistry	Result	Reference range
Blood urea nitrogen	19	9-25 mg/dL
Uric acid	10.1	3.5-8.5 mg/dL

He was admitted to the intensive care unit (ICU) due to the risk of leukostasis. Hematology-Oncology was consulted, and he was managed with one session of leukapheresis with improved WBC level of 235.56 x 10^3^/uL, followed by hydroxyurea 1000 mg twice daily and allopurinol 300 mg daily. His HIV RNA viral load was 40 copies/mL, and ART was continued. Uric acid was monitored and remained within normal limits during hospitalization, and cardiology, nephrology, and psychiatry services were also involved in the monitoring.

**Figure 1:**
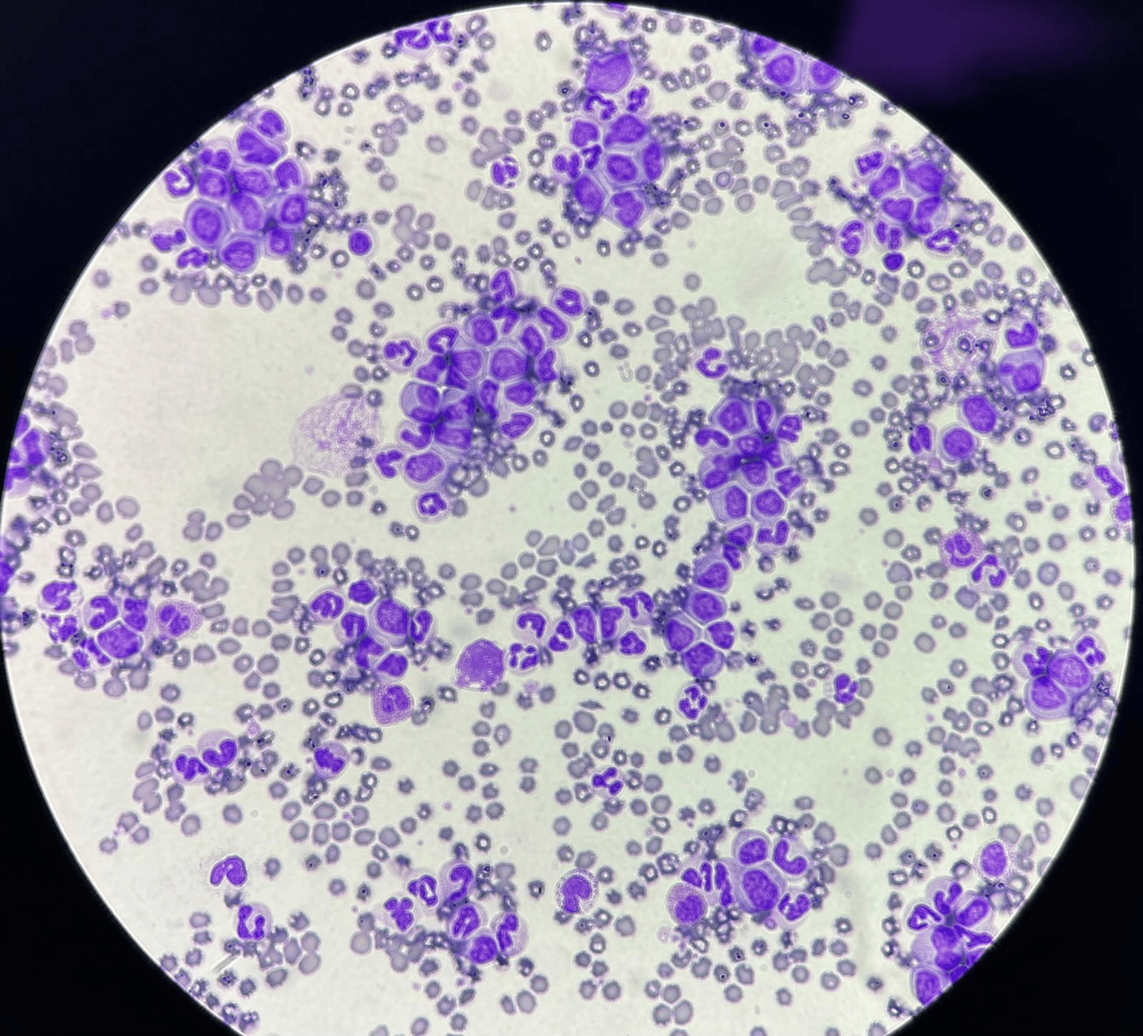
Peripheral blood smear: predominance of myeloid cells with large nuclei and a high nucleus-to-cytoplasm ratio.

The patient reported being diagnosed with CML four years prior, his last evaluation was three years prior, and his baseline WBC count was typically around 220 x 10^3^/uL. The peripheral blood smear showed a predominance of myeloid cells with features suggesting immaturity with large nuclei and a high nucleus-to-cytoplasm ratio (Figure 1).

Bone marrow aspiration confirmed BCR-ABL positivity, and flow cytometry of peripheral blood showed increased myeloblasts and immunophenotypically abnormal granulocytes and monocytes, with 1.5% blasts positive for CD13 (partial), CD33, CD34, CD38, CD117, HLA-DR, and negative for cCD3, cCD79a, MPO, and TdT.

Bone marrow cytometry showed immunophenotypically abnormal granulocytes and monocytes, no evidence of acute leukemia or lymphoproliferative neoplasm, abnormal CD56 and CD64 expression in granulocytes, and abnormal CD56 expression in monocytes. 

Following confirmation of CML, arrangements were made to start dasatinib, a tyrosine kinase inhibitor. The patient continued on hydroxyurea and allopurinol under close observation, resulting in a reduction of WBC count to 28.80 x 10^3^/uL upon discharge. 

## Discussion

Up to 50% of CML patients may be asymptomatic at diagnosis or may present symptoms like anemia-related fatigue, weight loss, gastrointestinal bleeding, gout, hyperleukocytosis, splenomegaly, or early satiety [7]. Severe hyperleukocytosis can result in leukostasis and impaired tissue perfusion, which may manifest as vascular occlusion symptoms, including central nervous system (CNS) abnormalities, respiratory distress, and organ failure, necessitating urgent treatment at symptom onset [8,9]. Our patient’s presenting symptoms, i.e., dizziness, blurry vision, and palpitations, are suggestive of leukostasis. 

CML diagnosis typically begins with complete blood count testing, but definitive confirmation requires bone marrow evaluation, including biopsy or aspiration with cytology, cytogenetics, and molecular testing to demonstrate BCR-ABL positivity consistent with the Philadelphia chromosome translocation [1,9,10]. In PWLH, assessment of HIV-1 RNA and CD4 cell count assesses the severity of the disease and therapy response. Chronic inflammation can persist despite antiretroviral control [11] with unknown interference with laboratory tests, such as HIV-1 RNA or flow cytometry. Our patient underwent a work-up, including viral load, CD4 counts, bone marrow biopsy, peripheral smears, and imaging, to develop a definitive care plan.

CML accounts for a substantial proportion of adult leukemias and has a notable incidence among older adults; it has also been reported to occur concurrently with HIV as a coincidental association, as in our patient [1,12]. Among patients with both HIV and CML, the success of ART has been associated with an increased incidence of hematologic malignancies, which can be explained because of prolonged survival [13]. Patients with CML-HIV often face a more aggressive disease due to immune dysregulation and the interaction between HIV and CML [9].

Concurrent use of ART and tyrosine kinase inhibitors (TKIs) can appropriately manage patients with CML-HIV, despite possible drug interactions [2]. Concurrent treatment of both conditions poses additional complexities, particularly regarding myelosuppression from both ART and tyrosine kinase inhibitors (TKIs). Careful management is essential, as both conditions and their treatment affect bone marrow function.

A central learning point from this case is that acute symptomatic hyperleukocytosis can be stabilized effectively while maintaining ART. Management of leukocytosis in CML, similar to acute myelogenous leukemia (AML), involves cytoreduction of the abnormally proliferating cell lines through leukapheresis [8], which quickly reduces WBC count by physically removing leukocytes, crucial in cases of leukostasis, and is usually reserved for symptomatic patients unable to start chemotherapy immediately. Hydroxyurea, on the other hand, is used to reduce hyperleukocytosis by ≥50% within 24 to 48 hours and is often used before definitive chemotherapy. Usually, patients unable to receive intensive induction therapy are started on hydroxyurea and leukapheresis rather than either therapy [8]. Our patient received one inductive therapy with leukapheresis and was started on hydroxyurea; meanwhile, arrangements were prepared to start the TKI medication. Multi-specialty input is essential for optimizing outcomes and minimizing complications in patients with CML-HIV. Supportive measures, like hydration and uric acid-lowering therapy, were used to reduce the risk of tumor lysis syndrome (TLS) and renal injury during rapid cytoreduction [14]. This combined approach was essential for stabilization and mitigation of early complications. 

Following stabilization, initiation of definitive CML therapy should not be delayed [15]. Selection of a first-line TKI is individualized based on disease risk, comorbidities, and toxicity profile. Importantly, many TKIs are metabolized via cytochrome P450 pathways (notably CYP3A4), which makes medication reconciliation and interaction screening a required step when patients are receiving ART [16,17]. Antiretroviral and TKI interactions require a tailored approach, and this may involve adjusting dosages or switching medications to ensure efficacy and minimize side effects. 

According to the literature, dasatinib treats CML as a potent TKI while boosting innate immunity by expanding memory-like NK cells and gamma delta T cells, which are linked to better control of both leukemia and HIV [18]. Dasatinib also showed anti-inflammatory properties [19] and may shrink the HIV latent reservoir by keeping SAMHD1 active to block new infections and by suppressing gamma c cytokine-driven homeostatic proliferation of infected cells [20]. While these mechanistic properties are of scientific interest, they were not measured in this case and do not directly inform the clinical decisions described here. Accordingly, the revised focus is on pragmatic bedside management.

PLWH with uncontrolled CML and symptomatic hyperleukocytosis can be successfully stabilized with leukapheresis and hydroxyurea while continuing ART, followed by timely initiation of definitive TKI therapy. Ongoing research on TKI-HIV interactions, especially with dasatinib, and collaboration among infectious disease specialists, oncologists, and pharmacists are key to improving care and quality of life for these patients [1]. Further studies are needed to better understand the long-term implications of dual therapy and to explore the potential benefits of TKIs beyond their oncological effects.

## Conclusions

Management of HIV and CML is well established individually, but their coexistence requires coordinated care to address infection risk, overlapping toxicities, and potential drug-drug interactions. This case highlights early recognition and prompt treatment of symptomatic hyperleukocytosis with leukapheresis and hydroxyurea, and shows that with modern ART, CML stabilization and transition to definitive TKI therapy can often proceed without interrupting ART when medication reconciliation and interaction screening are performed. Multidisciplinary collaboration is essential, and while dasatinib may be a valuable ally in CML-HIV co-management given emerging immunomodulatory data, these mechanisms were not evaluated in our patient and are not the focus of this report. 
